# Real-Time Ozone Sensor Based on Selective Oxidation of Methylene Blue in Mesoporous Silica Films

**DOI:** 10.3390/s19163508

**Published:** 2019-08-10

**Authors:** Christelle Ghazaly, Marc Hébrant, Eddy Langlois, Blandine Castel, Marianne Guillemot, Mathieu Etienne

**Affiliations:** 1 Département de Métrologie des Polluants, Institut National de Recherche et de Sécurité (INRS), 54500 Vandœuvre-lès-Nancy, France; 2 Laboratoire de Chimie Physique et Microbiologie pour les Matériaux et l’Environnement (LCPME), UMR 7564 CNRS-Université de Lorraine, 405 rue de Vandœuvre, 54600 Villers-lès-Nancy, France

**Keywords:** ozone, optical gas sensor, mesoporous silica, methylene blue, occupational exposure

## Abstract

Sensitive and selective personal exposure monitors are needed to assess ozone (O_3_) concentrations in the workplace atmosphere in real time for the analysis and prevention of health risks. Here, a cumulative gas sensor using visible spectroscopy for real-time O_3_ determination is described. The sensing chip is a mesoporous silica thin film deposited on transparent glass and impregnated with methylene blue (MB). The sensor is reproducible, stable for at least 50 days, sensitive to 10 ppb O_3_ (one-tenth of the occupational exposure limit value in France, Swiss, Canada, U.K., Japan, and the USA) with a measurement range tested up to 500 ppb, and insensitive to NO_2_ and to large variation in relative humidity. A model and its derivative as a function of time are proposed to convert in real time the sensor response to concentrations, and an excellent correlation was obtained between those data and reference O_3_ concentrations. This sensor is based on a relatively cheap sensing material and a robust detection system, and its analytical performance makes it suitable for monitoring real-time O_3_ concentrations in workplaces to promote a safer environment for workers.

## 1. Introduction

Ozone (O_3_) is a powerful oxidizing gas and a strong disinfectant used in different processes, including water treatment, gas purification, textile bleaching, and food industries. In workplaces, O_3_ is mainly emitted into the atmosphere from the processes of UV radiation and electric arc welding [1]. Another source of exposure in tertiary and service sectors is the extensive use of laser printers and photocopiers [2].

In France, the occupational exposure limits values (OELs) for O_3_ exposure are 100 ppb and 200 ppb for exposure times of 8 h and 15 min, respectively [3]. These values are consistent with other European or international OELs (see Table S1 in Supporting Information). At an O_3_ concentration exceeding OELs, exposure becomes hazardous to human health, causing headache, burning eyes, lung damage, and respiratory diseases such as asthma [4]. Therefore, it is necessary to monitor O_3_ concentrations in workplaces, using a personal monitor that provides real-time measurements of O_3_ exposure levels with high resolution in both time and concentration. Currently, chemical analysis techniques based on individual sampling on solid adsorbents or filters are used to determine concentrations of O_3_ [5,6,7,8,9], but delayed analysis of the samples in chemical laboratories is required. These techniques do not provide an alert in real time in the case of high exposure. Moreover, the obtained data are only average concentrations over extended periods.

Recently, numerous O_3_ sensors have been developed for incorporation into personal O_3_ samplers. For example, conductivity sensors based on a heated metal oxide semiconductor (HMOS), including ZnO, WO_3_, SnO_2_, In_2_O_3_, NiO, and CuO [10,11,12,13,14,15,16], have been used to detect O_3_. The majority of these sensors showed high stability of the baseline and fast response and recovery time with total reversibility. However, O_3_ sensors based on HMOS suffer from a lack of selectivity in the presence of other gaseous pollutants such as NO_2_ [17,18].

Electrochemical sensors have also been employed for monitoring air quality in occupational and environmental health applications [19]. They are characterized by their low cost, light weight, and high O_3_ sensitivity in the range of 5 ppb to 10 ppm. However, changes in relative humidity (RH) can generate a significant variation in the signal of the sensor [20]. Besides this, cross-sensitivity with NO_2_ remains a major problem to eliminate in the case of electrochemical O_3_ sensors [21].

Gravimetric sensors based on a quartz crystal microbalance (QCM) coating with 1,4-polybutadiene have shown good performance for indoor O_3_ applications [22]. The polybutadiene–QCM exhibits an irreversible reaction with O_3_ with a detection limit below 10 ppb [23] and insignificant interference from NO_2_, formaldehyde, CO, and phenol. However, polymer-coated quartz was found to be unstable with time [24].

Several UV-based O_3_ monitors have been developed, such as the 2B Technology 205 Dual Beam Ozone Monitor [25] and the Thermo Scientific Model 49i [26]. These instruments exhibit high sensitivity, selectivity, and reliability. However, the size, cost, and regular calibration needs prevent their use for real-time measurements when portability of the equipment is required. A highly portable O_3_ monitor called the Personal Ozone Monitor (POM) was recently developed by 2B Technologies [27]. This device based on UV absorbance exhibits accurate (≤2 ppb) O_3_ measurements and a detection limit of 4.5 ppb. However, the POM detects O_3_ concentrations less precisely in the presence of other pollutants absorbing in the UV domain, such as toluene, ethylbenzene, and xylenes, and for that reason it would be interesting to perform ozone measurements in the visible wavelength range [28].

Various chemiluminescent sensors have been developed for measuring O_3_ concentrations in ambient air. Most of these devices are based on the light-emitting reaction of O_3_ in the presence of an excess of ethylene or NO gas [29,30]. They present a detection limit of 1 ppb, but these devices require a continuous flow of gas supply from a pressurized cylinder, which limits the portability of these instruments. On the other hand, a continuous monitoring system for O_3_ based on chemiluminescent dye solution has been used. It utilizes ethanol solutions of rhodamine B and gallic acid bubbled with ozonized air. Rhodamine B shows a high specificity for O_3_ and a detection limit in the ppm range. However, the high level of noise resulting from the bubble systems is a drawback to using this method for personal monitoring [31].

Besides these, simple and compact O_3_ sensor devices have been developed based on dye fading colorimetry [32]. These sensors use materials that fade upon reaction with O_3_ and whose concentration can be quantified by absorbance or reflectance spectroscopy, such as curcumin and indigo carmine [33,34,35]. An accumulative and passive O_3_ sensor was proposed by Yasuko Yamada [36] based on porous glass impregnated with indigo carmine. The detection limit of this sensor chip is 3 ppb for a 1 h exposure, but cross-sensitivity towards 10 ppb of NO_2_ was observed.

**Figure 1:**
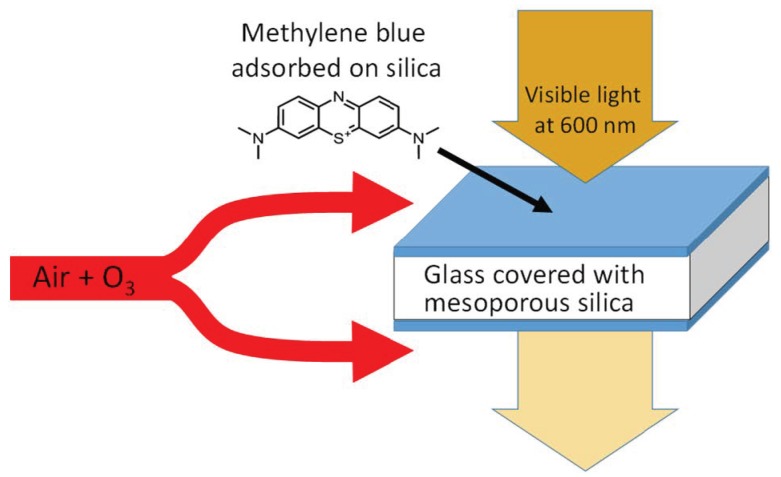
Principle of the sensor. A glass slide is covered on both sides with a mesoporous silica thin film on which methylene blue is adsorbed. Visible light at 600 nm is absorbed by this colored material. Exposure to O_3_ in the atmosphere leads to an immediate degradation of the dye. The kinetic of absorbance decrease is immediately translated to a concentration.

Our aim is to develop an affordable, simple, and portable sensor for O_3_ monitoring in workplaces, characterized by high sensitivity and selectivity in the presence of other gaseous pollutants. For that purpose, we chose methylene blue (MB), a blue cationic dye that belongs to the phenothiazine family. In previous studies, MB was used to monitor humidity [37,38,39]. The degradation of MB in aqueous solution by O_3_ was studied by Zhang et al. [40] and by Al Jibouri et al. [41]. In addition, MB dye immobilized on Nafion^®^ film was used for the detection of hydroxyl radicals in the atmosphere [42] but not applied to O_3_ monitoring. In this work, a real-time O_3_ sensor is elaborated by using visible spectroscopy as a measurement method and MB adsorbed on mesoporous silica thin film as the sensing material (Figure 1). A test bench was developed and implemented to produce controlled atmospheres for sensor evaluation. The sensor stability, sensitivity, and selectivity to O_3_ were investigated. Finally, a model and its derivative as a function of time were evaluated to convert in real time the sensor response to a concentration.

## 2. Materials and Methods

### 2.1. Chemicals and Reagents

All chemicals were used without further purification: tetraethoxysilane (TEOS, 98%, Alfa Aesar, Thermo Fisher, kandel, Germany), cetyltrimethylammonium bromide (CTAB, 99%, Acros, Thermo Fisher Scientific, Geel, Belgium), absolute ethanol (C_2_H_5_OH, 99.8%, Sigma-Aldrich, Merck, St. Quentin Fallavier, France), and hydrochloric acid (HCl, 36%, Prolabo, VWR, Paris, France) were used for the preparation of mesoporous silica films on glass microscope slides (Rogo Sampaic) previously washed with sodium hydroxide (NaOH, 1 M, Alfa Aesar, Thermo Fisher, kandel, Germany). Methylene blue (>82%, Fluka, Fisher Scientific, Illkirch, France) was used as the O_3_ sensing material. All solutions were prepared with high-purity water (18 MΩ.cm) from a Millipore Milli-Q^®^ water purification system (Millipore SAS, Molsheim, France). 

### 2.2. Sensor Chip Preparation

Mesoporous silica was prepared with CTAB as the surfactant template and according to the procedure previously reported by Etienne et al. [43]. The initial solution was prepared as follows: A quantity of 0.51 g of CTAB was dissolved in 0.9 g of water and 11.66 g of ethanol with stirring for 30 min at room temperature. Then, 2.23 g of TEOS was slowly added into that solution. After that, 0.04 ml of HCl (1 mol L^−1^) was directly added to the mixture. The final reactant molar ratios were 1TEOS/20C_2_H_5_OH/5H_2_O/0.004HCl/0.14CTAB. The solution was aged at room temperature in sealed vessels for three days before deposition on glass plates of 1 mm thickness. Prior to use, substrates were shaped into squares of 8 mm × 9 mm and washed successively with a 1 mol L^−1^ solution of NaOH and deionized water. The mesoporous silica film was deposited on glass by dip-coating at a withdrawal speed of 2.5 mm s^−1^ and under 50% RH. Then, the film was stabilized at 130 °C for 48 h and calcined at 450 °C for 5 h before use. Figure S1 reports the typical mesostructure that was achieved. In addition, the film thickness determined by profilometry was 181 ± 11 nm (*N* = 8) (Table S2). Methylene blue was absorbed on mesoporous silica by immersion for 1 min into a 3 mmol L^−1^ solution of MB, followed by rinsing with deionized water. After drying at room temperature, a blue film was obtained. The sensing chips were stored in plastic vessels laminated with aluminum.

### 2.3. Experimental Setup

**Figure 2:**
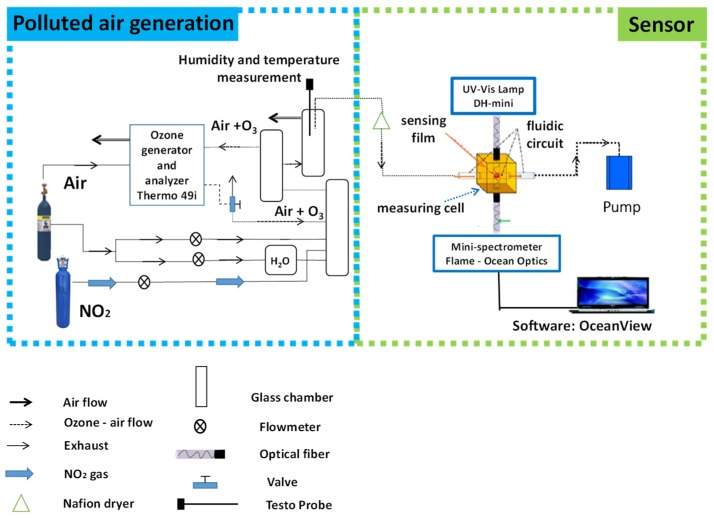
Schematic diagram of the experimental setup.

The setup used in this work is detailed in Figure 2. It was composed of a polluted air generator connected to the sensor. Gas mixtures containing different O_3_ concentrations were prepared in a cylindrical glass chamber (1.5 L in volume). O_3_ was generated by passing a flow of dry air through an O_3_ generator (Thermo Scientific Model 49*i* UV Photometric O_3_ Analyzer); the O_3_ monitor was also used for the control of O_3_ concentrations in all experiments. Two mass flow controllers were used to dilute O_3_ gas in purified air in order to achieve lower O_3_ concentrations (10–100 ppb) in order to be in the range of the concentrations usually measured in workplaces. Humidification to various degrees was controlled by bubbling an adjustable portion of the dilution air through a water bubbler. A Testo435 portable sensor was placed in the glass chamber to monitor the temperature and the relative humidity. The developed sensor was inserted into a 8 mm width and 1 mm thickness slot within a brass measuring cell [44] under different O_3_ concentrations. A pump was used to ensure continuous flow of O_3_ between 50 and 400 mL min^−1^ inside the measuring cell. The pump was connected to the sampling cell with a Teflon tube. The measuring cell was exposed continuously to visible light emitted from a DH-mini Ocean Optics source. A portable Ocean Optics Flame mini-spectrometer with optical fiber was employed to measure in situ material absorption at a fixed wavelength during the O_3_ exposure trials. the absorption signal was recorded using OceanView spectroscopy software. An interference study with NO_2_ was carried out using the same experimental setup previously described. Known concentrations of NO_2_ were directly generated from a calibration gas cylinder (GasDetect, 27 ppm) and delivered to the mixing glass chamber via Teflon tubing. In addition, a calibrated mass flow controller was used to dilute NO_2_ in purified air.

### 2.4. Mathematical Model

A simple mathematical model was applied to fit with the experimental results by using Equation (1) derived from the pseudo-second-order kinetic equation [45]. This equation takes into account both the rate-limited surface reaction and mass transfer by diffusion in a complex medium:

(1) $$A_{t}=\left[\frac{\left(A_{t_{0}}-A_{inf}\right)}{\left(1+k_{0}\left[O_{3}\right]t\left(A_{t_{0}}-A_{inf}\right)\right)}\right]+\;A_{inf}$$

where $A_{t}$ represents the absorbance at sampling time t (min); $A_{t_{0}}$ is the initial absorbance at initial time $t_{0}$ (min); $A_{inf}$ is the absorbance after an infinite O_3_ sampling time; $\left[O_{3}\right]$ represents the ozone concentration measured by the O_3_ analyzer (ppb); and $k_{0}$ is the rate constant of the reaction (ppb^−1^ min^−1^).

Moreover, the model previously presented was also applied to predict absorbance variations during O_3_ exposure at different concentrations. In this case, Equation (2) was used, where $A_{calc}$ represents the calculated absorbance at sampling time *t*.

(2) $$A_{calc}=\left[\frac{\left(A_{t_{0}}-A_{inf}\right)}{\left(1+k_{0}\left(t-t_{0}\right)\left(A_{t_{0}}-A_{inf}\right)\left[O_{3}\right]\right)}\right]+\;A_{inf}$$

Additionally, the O_3_ concentration detected by the developed sensor was calculated by applying Equation (3):

(3) Csensor=[(At0−At)(At−Ainf)k0(At0−Ainf)(t−t0)]

where $C_{sensor}$ represents the O_3_ concentration detected by the sensor (ppb).

Finally, we also evaluated the application of the derivative as a function of time (Equation (4)) for concentration determination.

(4) $$\frac{\partial \left(A\left(t\right)\right)}{\partial t}=\frac{-k_{0}{\left(A_{t_{0}}-A_{inf}\right)}^{2}C_{sensor}}{{\left(1+k_{0}\left(A_{t_{0}}-A_{inf}\right)\left(t-t_{0}\right)C_{sensor}\right)}^{2}}$$

## 3. Results and Discussion

### 3.1. Preliminary Studies

**Figure 3:**
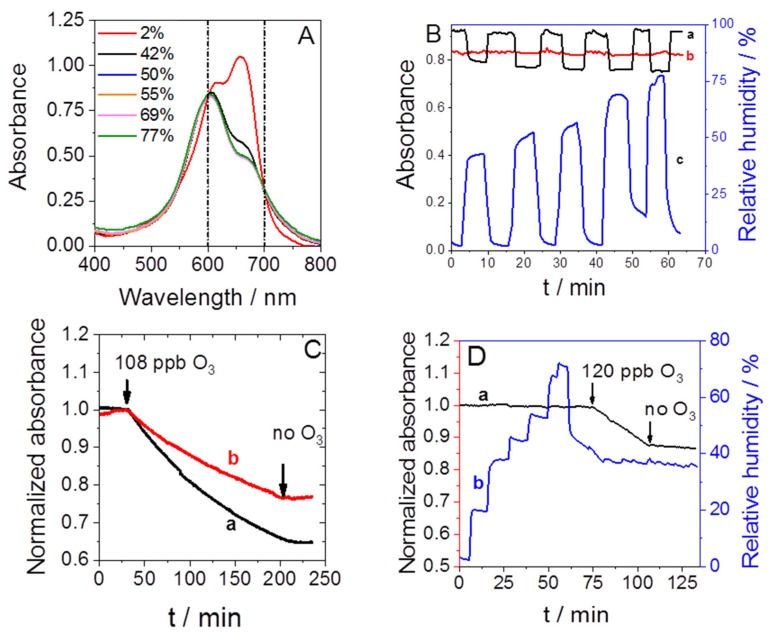
(**A**) Absorbance of methylene blue in the mesoporous silica in the presence of 2%, 42%, 50%, 55%, 69%, and 77% relative humidity in air. (**B**) Absorbance of methylene blue in the mesoporous silica measured as a function of time at 620 nm (a, black) and 600 nm (b, red) while the relative humidity varied from 2% to 77% (c, blue). (**C**) Variation of the normalized absorbance in the presence of 108 ppb O_3_ as a function of time in air with 1% (a) or 40% RH (b). (**D**) Sensor response as a function of time, relative humidity, and O_3_ concentration in air (a), and the amplitude of the relative humidity variation as a function of time during the experiment (b).

The visible spectra of methylene blue adsorbed in mesoporous silica show two characteristic absorption peaks of MB at 620 and 665 nm (Figure 3A), similar to the literature [38]. Preliminary tests were performed to evaluate the sensor response as a function of humidity. When the relative humidity was varied between 2% and 77%, a significant decrease in absorbance was observed at these two wavelengths (Figure 3A), in agreement with previous reports [46]. On the contrary, the absorbance increased at 560 nm and at wavelengths higher than 700 nm. Two isosbestic points were found at 600 and 700 nm for which negligible absorbance variation was observed when the humidity was changed. Figure 3B demonstrates the critical influence of the wavelength on the sensitivity to humidity (see curve (c) for the amplitude of the relative humidity in this experiment). While the absorbance changed dramatically when the measurement was done at 620 nm (curve (a)), only limited variations in the absorbance were observed at 600 nm (curve (b)). So, it is possible, by working at 600 nm, to follow the absorbance of methylene blue in the presence of variable relative humidity.

However, humidity also affects the reaction rate of ozone with methylene blue. In another preliminary experiment done at 620 nm and at 50 mL min^-1^, the sensor was exposed to 108 ppb of O_3_ in the presence of 1% (Figure 3C, curve (a)) or 40% relative humidity (Figure 3C, curve (b)) for almost three hours. The sensor detected O_3_ in both conditions, but the absorbance decreased more rapidly in dry air than in humid air. We can model the sensor response with Equation (1), and the rate constant of MB discoloration (k_0_) allows a quantitative assessment of its sensitivity. In a humid atmosphere, the rate constant is 5.61 × 10^−5^ ppb^-1^ min^−1^, while it is almost 2 times higher in dry air at 1.08 × 10^−4^ ppb^−1^ min^−1^ (note that discoloration was homogenous over the all absorbance spectra of MB, so the conclusion of this set of experiments does not depend on the wavelength of measurement). The sensitivity of the sensor was achieved thanks to the reaction between MB adsorbed on porous silica and reactive oxygen species produced from O_3_ molecules diffusing inside the mesopores of the thin film. The mechanism involves the production of hydroxyl radicals [47]. Tertiary amines are converted to primary amines, and the central ring of the native molecule opens [48]. These reactions are probably responsible for the irreversible discoloration. At 40% relative humidity, the mesopores are partially filled with water [49] which slows down the transfer of the reactive species and modifies the conditions of these reactions. Two strategies can be considered at this step of the sensor development. First, absorbance and air humidity can be monitored simultaneously in order to adjust in real time the sensitivity of the sensor given by k_0_ during the measurement, so as to determine the O_3_ concentrations. The second strategy that we applied in this work is to significantly decrease the relative humidity in the air before analysis. This can be achieved by using an efficient gas dryer between the sampling source and the detection cell (see the Supplementary Information associated with Figure S2). Air containing 50% to 75% RH (Figure S2, curve (a)) was thereby dried to a level of humidity close to 5% (5.5 ± 1.6) in a single pass (Figure S2, curve (b)).

We applied this air drier in the following O_3_ exposure trials using a sampling rate of 350 mL min^−1^, and furthermore, all measurements were done at the wavelength of 600 nm, which is not sensitive to minor variations in relative humidity. Figure 3D reports one illustrative experiment, in which the sensor was exposed to various relative humidity levels between 2% and 72% before exposure for a duration of 30 min to 120 ppb of O_3_ at about 36% relative humidity. The normalized absorbance was stable as a function of time when the humidity was changed dramatically and decreased only when ozone was present in the analyzed air (first arrow at 74 min); it stopped decreasing when O_3_ was not present (second arrow at 106 min).

**Figure 4:**
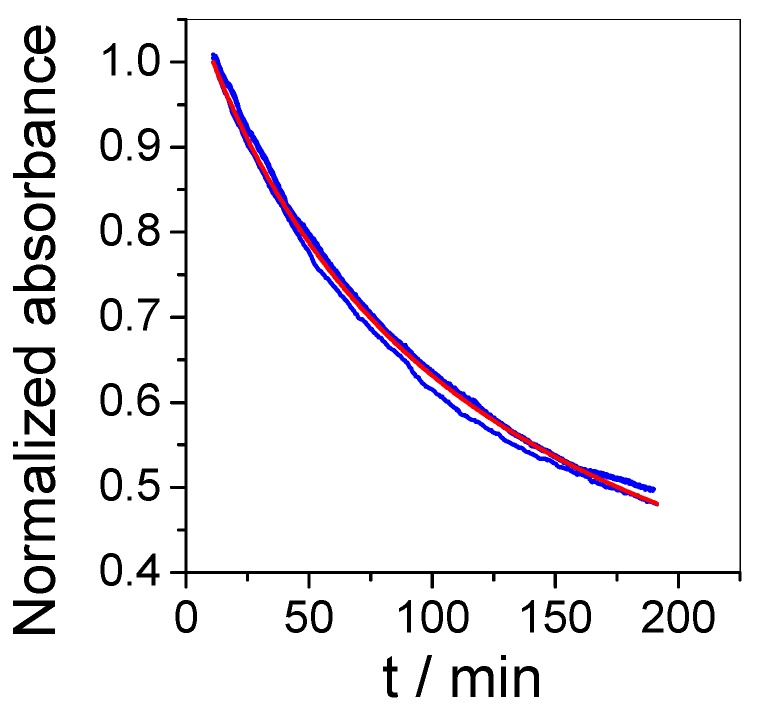
Experimental (blue) and model (red) curves of the normalized absorbance as a function of time in the presence of 120 ppb for three sensors analyzed at Day 1, Day 2, and Day 50 (k_0_ = 8.1 × 10^−5^ ppb^−1^ min^−1^ and A_inf_ = 0.137 ± 0.003).

The response to O_3_ was reproducible and stable with time, as illustrated in Figure 4 which reports three measurements made in the presence of 120 ppb ozone and 36% relative humidity for three hours with sensors prepared from the same batch and analyzed at Days 1, 2, and 50. All measurements show good correlation with the model curve calculated using Equation (2) considering k_0_ = 8.1 × 10^−5^ ppb^−1^ min^−1^.

### 3.2. Sensor Sensitivity and Selectivity

**Figure 5:**
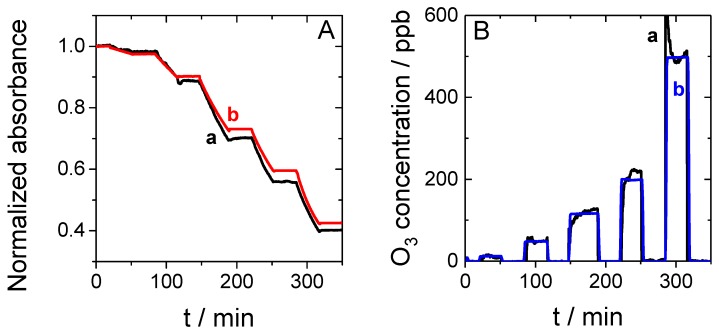
(**A**) Experimental (a, black) and model (b, red) curves showing the normalized absorbance as a function of time in the presence of increasing concentrations of O_3_ from 12 to 500 ppb. (**B**) O_3_ concentration measured as a function of time with the O_3_ sensor (a, black) and controlled values given by a benchtop O_3_ analyzer (b, blue).

The performance of the sensor was then evaluated in the presence of O_3_ concentrations ranging from 10 to 500 ppb. Figure S3 reports the gradual discoloration of the sensor chip composed of MB adsorbed on a mesoporous silica thin film when it was exposed to O_3_. Only a decrease of absorbance was observed, and no other absorption peak appeared in the visible wavelength window during O_3_ exposure. Figure 5A reports the evolution of the normalized absorbance as a function of time while the O_3_ concentration was varied stepwise from 10 to 500 ppb in the presence of 36% relative humidity.

Each exposure lasted 30 min and was followed by 30 min without O_3_. The absorbance decreased in the presence of O_3_ and did not vary in the absence of O_3_. Curve (a) presents the experimental data and curve (b) shows the model derived from Equation (2). Moreover, we evaluated the possibility to determine the concentration of O_3_ directly from the sensor response using Equation (3). Figure 5B reports the comparison of the sensor response as a function of time with the controlled concentration given by the O_3_ analyzer (see Figure 2 to visualize the position of this analyzer versus the sensor in the setup). As can be observed, a good correlation was found between the generated O_3_ concentration (curve (b)) and the sensor response (curve (a)) up to 10 ppb, i.e., far below the OEL for O_3_, which is 100 ppb for 8 h monitoring and 200 ppb for 15 min monitoring. In conclusion, the sensor provides sufficient sensitivity and measurement range (tested here up to 500 ppb) for O_3_ monitoring in working environments. Moreover, it provides a rapid warning for the exposed worker and temporally resolved data on O_3_ concentrations.

In addition to sensor sensitivity, it is important to evaluate cross-sensitivity resulting from gaseous pollutant compounds. The measurement of O_3_ in occupational environments is mainly affected by other oxidizing gases such as NO_2_ and sulfur dioxide (SO_2_) [50,51]. In the following interference testing, the cross-sensitivity versus NO_2_ was evaluated. Experiments were carried out by exposing the sensors to 510 ppb of NO_2_. The selected NO_2_ concentration is based on the OEL fixed in the recommendation for NO_2_ by the Scientific Committee on Occupational Exposure Limits (SCOEL) [52].

**Figure 6:**
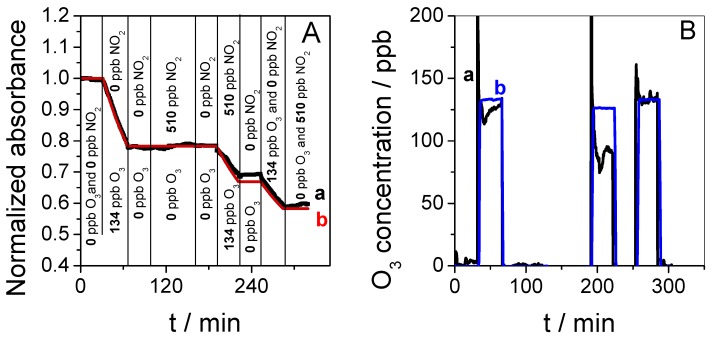
(**A**) Experimental (a, black) and model (b, red) curves showing the normalized absorbance as a function of time in the presence of 0 or 134 ppb of O_3_ and 0 or 510 ppb NO_2_. (**B**) O_3_ concentration measured as function of time with the O_3_ sensor (a, black) and controlled values given by a benchtop O_3_ analyzer (b, blue). The test was performed at 24 °C, with air at 50% RH passing through the Nafion^®^ tube at a flow rate of 350 mL min^-1^ and using a sampling time of 30 min.

Figure 6A shows the normalized absorbance variation at 600 nm as a function of time when the sensor was exposed to 134 ppb of O_3_ followed by exposure to 510 ppb of NO_2_ and, finally, to a mixture of 134 ppb O_3_ and 510 ppb NO_2_. The sensor showed a significant response during each exposure to O_3_ (1st at 30 min, 2nd at 190 min, and 3rd at 250 min). Oppositely, no response was observed when the sensor was exposed to 510 ppb from 100 to 160 min and after 294 min. Thus, NO_2_ did not interfere in the detection of O_3_.

Figure 6B reports the O_3_ concentration determined with Equation (3) during this complex scenario. The ozone concentration measured by the sensor (curve (a)) and the concentration measured by an O_3_ analyzer (curve (b)) fitted well when O_3_ was introduced alone in air. However, when 134 ppb O_3_ was generated in the presence of 510 ppb NO_2_, the concentration determined at the sensor was close to 90 ppb. Interestingly, the concentration determined with the O_3_ analyzer was also lower than expected—only 125 ppb was measured. In the gas mixture, the NO_2_ concentration was 5 times larger than that of O_3_. In these conditions, the reaction between the two molecules induces the formation of NO_3_ and N_2_O_5_ according to Reaction 1 followed by Reaction 2 [53,54].

$$\begin{array}{cc} {\mathrm{NO}}_{2}+O_{3}\to {\mathrm{NO}}_{3}+O_{2} & \;(1)\\ {\mathrm{NO}}_{3}+{\mathrm{NO}}_{2}⇌N_{2}O_{5} & \;(2)\\ 2{\mathrm{NO}}_{2}+H_{2}O\to H{\mathrm{NO}}_{3}+H{\mathrm{NO}}_{2} & \;(3) \end{array}$$

Furthermore, the presence of HNO_3_ resulting from Reaction 3 between NO_2_ and humidity is also possible. As a result, the initial O_3_ concentration generated is partly consumed by the formation of other gaseous species. In the experimental setup reported in Figure 2, the O_3_ analyzer was positioned in the fluidic system far before the sensor, and it is very possible that the real O_3_ concentration measured by the sensor is lower than that measured by the O_3_ analyzer because of the additional time needed for gas transfer leading to higher reaction progress before detection. To conclude, NO_2_ was not detected by the sensor based on methylene blue in mesoporous silica thin film, and the accuracy of the determination of O_3_ in the presence of NO_2_ depends essentially on the fluidic length between the sampling source and the detector, which must be as short as possible.

### 3.3. Application of the Sensor in a Complex Scenario

**Figure 7:**
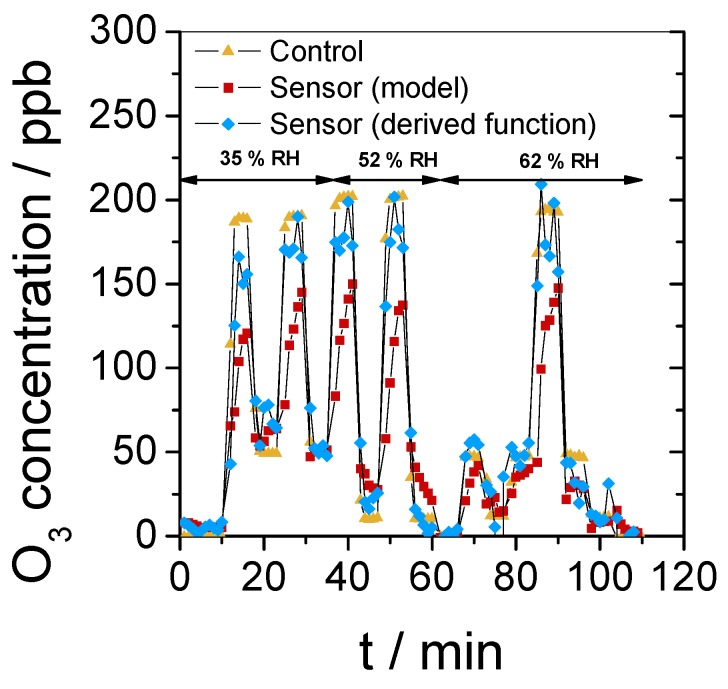
O_3_ concentration measured as function of time with a benchtop O_3_ analyzer for control (triangle) and the sensor using Equation (3) (square) or the derivative as a function of time (Equation (4), lozenge). In this scenario, the O_3_ concentration was varied every five to ten minutes from 0 to 10 or 50 or 200 ppb, and the relative humidity in air was changed from 35% to 52% and, finally, 62%.

To finalize this study, we evaluated the sensor in a more complex scenario during which both relative humidity (from 35% to 52% and 62%) and O_3_ concentration (from 10 to 200 ppb) were varied every five- to ten-minute period for almost two hours (Figure 7). The normalized absorbance profile of this experiment and the model absorbance curve, calculated according to Equation (2), can be found in Figure S4. The sensor response was first determined by applying Equation (3) to the experimental absorbance data and then compared to the concentration given by the O_3_ analyzer used here as a control. The low concentrations of O_3_, below 60 ppb, were well determined, but a significant difference was observed between the expected concentration (control) and the sensor response at this relatively short time scale, resulting in an error of about 30%. The reason for this could be that the time requested to reach a constant value of O_3_ concentration in the measuring setup is not negligible when a short exposure time is used. Oppositely, concentration steps of 30 min were accurately determined (Figure 5 and Figure 6). Moreover, Equation (3), used to convert absorbance to concentration, is potentially sensitive to a cumulative shift in the absorbance value, leading then to errors in the determination of O_3_ concentrations. These limits were overpassed by treating data with the derivative as a function of time, given in Equation (4). This method allows us to achieve rapid determination of the concentration that was introduced in air (see the blue triangle curve in Figure 7) and to prevent cumulative errors. Finally, this experiment confirmed that the water content in air from 35% to 62% relative humidity does not produce any interference in the O_3_ determination. We tested the derivative of the absorbance as a function of time for the treatment of data collected with different concentrations of O_3_ (Figure S5) and in the presence of NO_2_ (Figure S6). Despite the large noise, essentially due to the data collection parameters that were not optimal, it is clear that the method allows rapid determination of concentrations in these different conditions.

## 4. Conclusions

In this study, a real-time optical O_3_ sensor based on methylene blue adsorbed in a mesoporous silica thin film was successfully developed. The device includes a Nafion^®^ dryer to eliminate the interference of water. At 600 nm, the sensor exhibited interesting performance: it showed significant sensitivity at low O_3_ concentrations up to 10 ppb, good reproducibility and stability up to 50 days in the absence of O_3_, and no direct interference by NO_2_. The derivative of the absorbance as a function of time was proposed to reach a fast and accurate response to changes in O_3_ concentration. The next step in this research is the miniaturization of the sensor for analytical performance evaluation in workplaces and real-time monitoring of O_3_ exposure that will contribute to a safer working environment. The sensor will operate at a wavelength close to 600 nm and will integrate air sampling in a portable device.

## Supplementary Materials

The following are available online at https://www.mdpi.com/1424-8220/19/16/3508/s1.
