# Supplementary material for: Real-Time Ozone Sensor Based on Selective Oxidation of Methylene Blue in Mesoporous Silica Films
Source: Sensors (Basel). 2019 Aug 10;19(16):3508. doi: 10.3390/s19163508 (PMC6720768; doi:10.3390/s19163508)
Supplement: Supplementary file 1 [file sensors-19-03508-s001.pdf]

Supporting information

Real-time ozone sensor based on selective oxidation of methylene blue in mesoporous silica films

Christelle Ghazaly[1, 2], Marc Hébrant[2], Eddy Langlois[1], Blandine Castel[1], Marianne Guillemot[1,*], Mathieu Etienne[2,*]

[1] Institut National de Recherche et de Sécurité (INRS), 54500 Vandœuvre-lès-Nancy, France.
[2] Université de Lorraine, CNRS, LCPME, F-54000 Nancy, France.
* Correspondence: marianne.guillemot@inrs.fr (M.G.); mathieu.etienne@univ-lorraine.fr (M.E.)

Figures and details

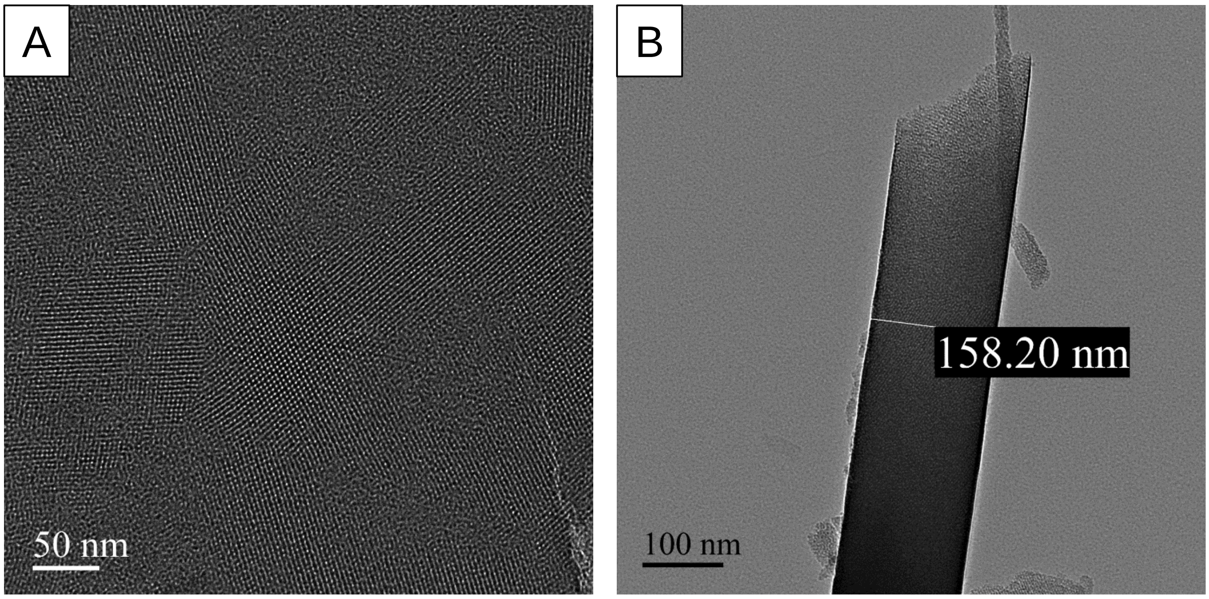

Figure S1. Transmission electron micrographs of a sample of mesoporous silica, (A) top view, (B) side view.

Transmission electron microscopy (TEM) investigations were carried out using a JEM - ARM 200F Cold FEG TEM/STEM operating at 200 kV and equipped with a spherical aberration (Cs) probe and image correctors (point resolution 0.12 nm in TEM mode and 0.078 nm in STEM mode).

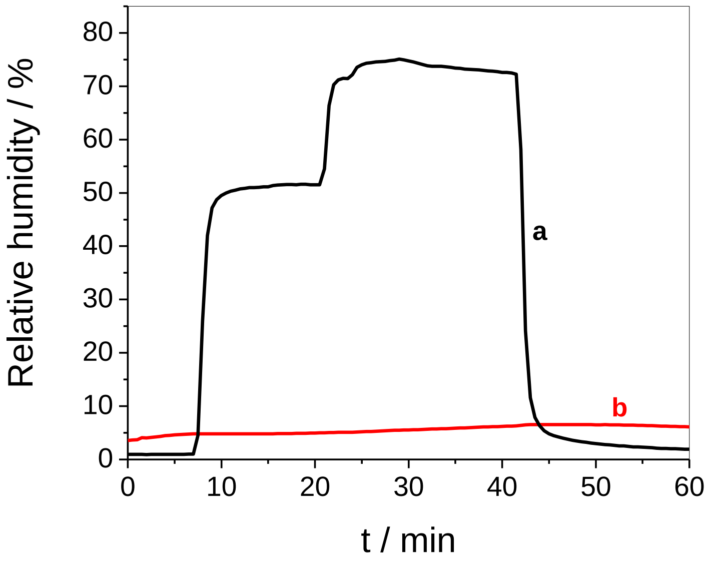

Figure S2. Relative humidity measured as a function of time in the inlet (a, black) and the outlet (b, red) of the air dryer. Nafion® is a copolymer of tetrafluoroethylene (Teflon®) and perfluoro-3, 6-dioxa-4-methyl-7-octene-sulfonic acid. Teflon makes it highly resistant to attack by strong oxidant gas such as O_3. In addition, the presence of sulfonic acid allowing absorption up to 13 molecules of water per sulfonate group makes the dryer highly performing in the removal of water[1]. We used in these experiments a monotube dryer of Nafion® (Perma Pure, Inc., MD-070-48F-2) housed within a single fluorocarbon large tube shell. The Nafion® tube was placed prior to the entrance of the measuring cell and was operated with the reflux method. The gas flow containing water vapor passes through Nafion® monotube, housed in a second tube which is continuously purged in countercurrent by the gas flow coming out of the measuring cell, at a rate three times higher than the sampling rate. The drying process is accomplished by the resulting gradient of the water vapor pressure between the inlet and the outlet of the Nafion® tube. In order to evaluate the drying performance of the system, dry air (1%) and humid air. (51% and 75%) was drawn through the Nafion® tube using a sampling flow rate of 400 mL min^{-1}. The humidity, before and after the dryer, was measured using two Testo humidity probes. Figure S4 shows that the RH of the outflow was equilibrated to 5.5 ± 1.6% for any variation of RH in the incoming flow. Therefore, the Nafion® dryer used shows the ability to equilibrate the humidity level of the sampling gas stream at about 6%.

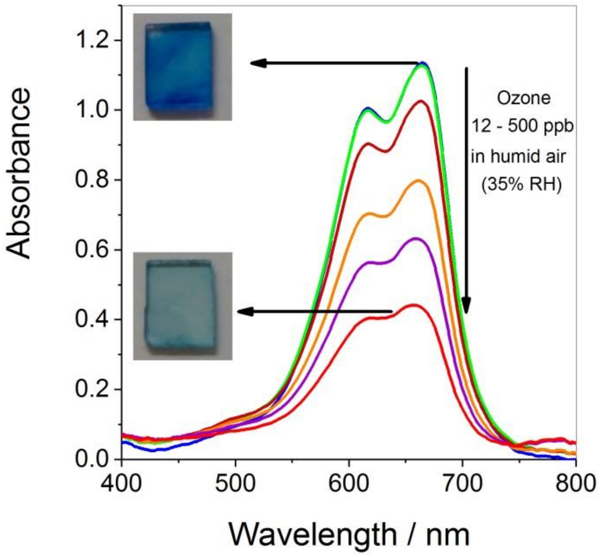

Figure S3. Evolution of the absorbance of visible light by methylene blue in mesoporous silica as a function of time in the presence of an increasing concentration of O_3 from 12 to 500 ppb. Inset images show the color of the sensor chip at the beginning and at the end of the experiment.

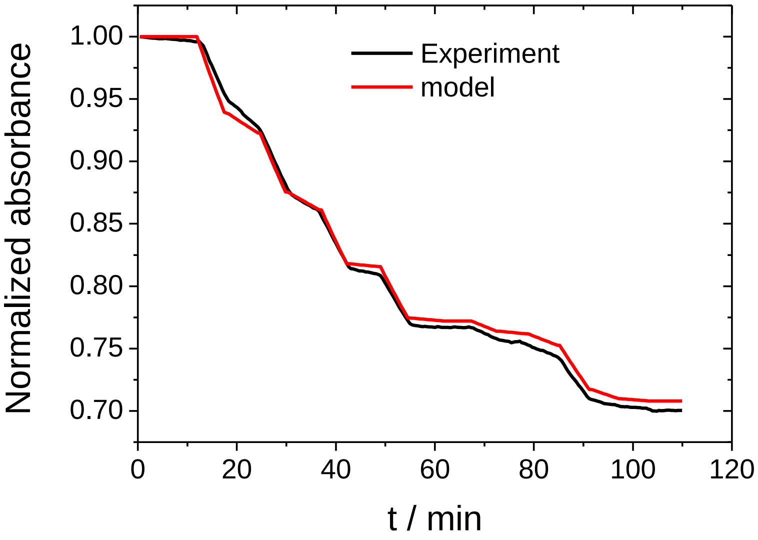

Figure S4. Experimental (black) and model (red) curves showing the normalized absorbance as a function of time while ozone concentration was varying every five to ten minutes from 0 to 10 or 50 or 200 ppb and the relative humidity in air was changing from 35 to 52 and finally 62%.

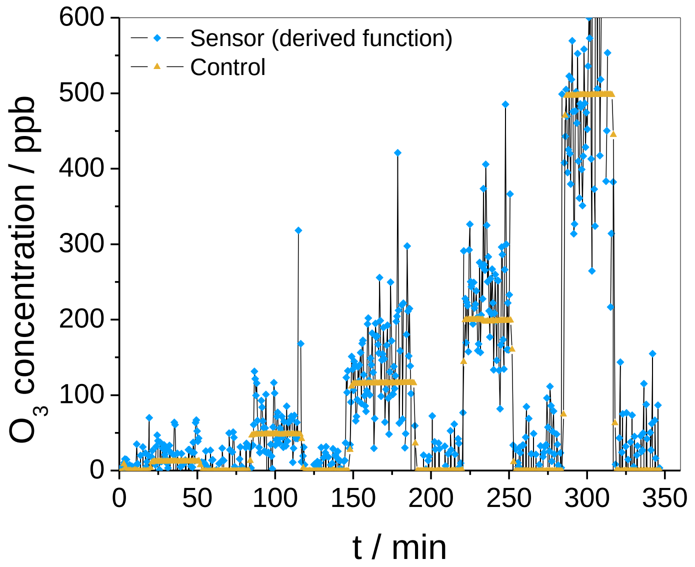

Figure S5. Ozone concentration measured as function of time in the presence of increasing concentrations of ozone from 12 to 500 ppb with the ozone sensor (blue lozenge, derivative as a function of time) and the controlled values given by a benchtop ozone analyzer Thermo 49i (orange triangle, control). Raw data identical as Figure 5.

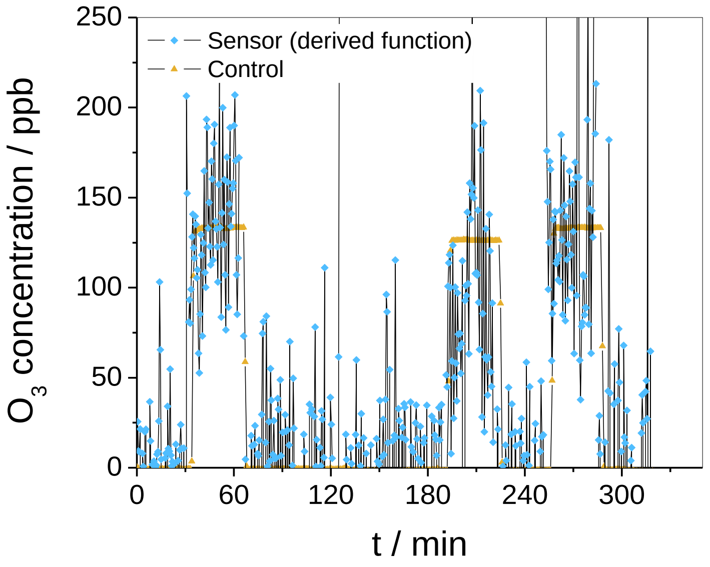

Figure S6. Ozone concentration measured as function of time in the presence of 0 or 134 ppb of O_3 and 0 or 510 ppb NO_2 with the ozone sensor (blue lozenge, derivative as a function of time) and the controlled values given by a benchtop ozone analyzer Thermo 49i (orange triangle, control). Raw data identical as Figure 6.

Tables

Table S1. Occupational exposure limit (OEL) values fixed for ozone in different countries.

Reference	Permissible Exposure Limit-Time Weighted Average (PEL-TWA: 8h) (ppm)			PEL-Short-Term Exposure Limit (PEL-STEL: 15-30 min) (ppm)
	Light work	*Moderate work*	*Heavy work*	
**OSHA (US)**	0.1	0.08	0.05	0.2
**WorkSafeBC (Canada)**	0.1	0.08	0.05	0.2
**ANSI/ASTM**	0.1	NA	NA	0.3
**ACGIH**	0.1	NA	NA	0.3
**NIOSH**	0.1	0.08	0.05	0.2
**INRS (France)**	0.1	NA	NA	0.2
**SUVA (Swiss)**	0.1	NA	NA	0.2
**JSOH (Japan)**	0.1	NA	NA	0.2
**HSE (UK)**	0.1	NA	NA	0.2

Table S2. Thickness values measured for mesoporous silica films synthesized. The determination of film thickness was carried out using the Bruker DektakXT profilometer.

	**Thickness (nm)**
**Film 1**	186.75
**Film 2**	180.86
**Film 3**	157.26
**Film 4**	185.20
**Film 5**	179.81
**Film 6**	193.88
**Film 7**	182.33
**Film 8**	184.76
**Average Thickness (nm)**	181.36
**Standard deviation**	10.67
